# Methyl (2*E*)-2-[(2,4-dioxo-1,3-thia­zolidin-3-yl)meth­yl]-3-phenyl­prop-2-enoate

**DOI:** 10.1107/S1600536812000578

**Published:** 2012-01-11

**Authors:** S. Vijayakumar, S. Murugavel, D. Kannan, M. Bakthadoss

**Affiliations:** a Department of Physics, Sri Balaji Chokkalingam Engineering College, Arni, Thiruvannamalai 632 317, India; b Department of Physics, Thanthai Periyar Government Institute of Technology, Vellore 632 002, India; c Department of Organic Chemistry, University of Madras, Maraimalai Campus, Chennai 600 025, India

## Abstract

In the title compound, C_14_H_13_NO_4_S, the thia­zolidine ring is essentially planar [maximum deviation = 0.010 (2) Å for the carbonyl C atom between the N and S atoms] and is oriented at a dihedral angle of 60.1 (1)° with respect to the benzene ring. In the crystal, mol­ecules are linked into zigzag chains running along the *c* axis by C—H⋯O hydrogen bonds. The crystal packing is further stabilized by C—H⋯π inter­actions involving the benzene ring.

## Related literature

For the biological activity of thia­zolidine derivatives, see: Chen *et al.* (2000 ▶); Jacop & Kutty (2004 ▶); Kalia *et al.* (2007 ▶); Vicentini *et al.* (1998 ▶); Vigorita *et al.* (1992 ▶). For resonance effects of acrylate, see: Merlino (1971 ▶); Varghese *et al.* (1986 ▶). For closely related structures, see: Fun *et al.* (2009 ▶); Vijayakumar *et al.* (2012 ▶).
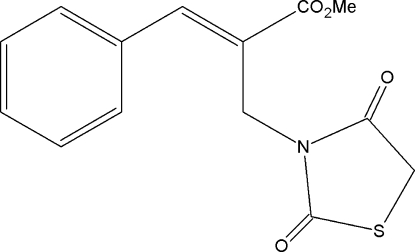


## Experimental

### 

#### Crystal data

C_14_H_13_NO_4_S
*M* *_r_* = 291.31
Orthorhombic, 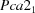

*a* = 11.9274 (3) Å
*b* = 15.6064 (6) Å
*c* = 7.2949 (3) Å
*V* = 1357.90 (8) Å^3^
*Z* = 4
Mo *K*α radiation
μ = 0.25 mm^−1^
*T* = 293 K
0.26 × 0.22 × 0.18 mm

#### Data collection

Bruker APEXII CCD diffractometer
Absorption correction: multi-scan (*SADABS*; Sheldrick, 1996 ▶) *T*_min_ = 0.937, *T*_max_ = 0.956
13845 measured reflections
2948 independent reflections
2576 reflections with *I* > 2σ(*I*)
*R*_int_ = 0.026

#### Refinement

*R*[*F* ^2^ > 2σ(*F* ^2^)] = 0.030
*wR*(*F* ^2^) = 0.082
*S* = 1.03
2948 reflections
182 parameters
1 restraint
H-atom parameters constrained
Δρ_max_ = 0.12 e Å^−3^
Δρ_min_ = −0.22 e Å^−3^
Absolute structure: Flack (1983 ▶), 1348 Friedel pairs
Flack parameter: 0.01 (7)

### 

Data collection: *APEX2* (Bruker, 2004 ▶); cell refinement: *APEX2* and *SAINT* (Bruker, 2004 ▶); data reduction: *SAINT* and *XPREP* (Bruker, 2004 ▶); program(s) used to solve structure: *SHELXS97* (Sheldrick, 2008 ▶); program(s) used to refine structure: *SHELXL97* (Sheldrick, 2008 ▶); molecular graphics: *ORTEP-3* (Farrugia (1997 ▶); software used to prepare material for publication: *SHELXL97* and *PLATON* (Spek, 2009 ▶).

## Supplementary Material

Crystal structure: contains datablock(s) global, I. DOI: 10.1107/S1600536812000578/bt5778sup1.cif

Structure factors: contains datablock(s) I. DOI: 10.1107/S1600536812000578/bt5778Isup2.hkl

Supplementary material file. DOI: 10.1107/S1600536812000578/bt5778Isup3.cml

Additional supplementary materials:  crystallographic information; 3D view; checkCIF report

## Figures and Tables

**Table 1:** Hydrogen-bond geometry (Å, °) *Cg* is the centroid of the C7–C12 benzene ring.

*D*—H⋯*A*	*D*—H	H⋯*A*	*D*⋯*A*	*D*—H⋯*A*
C2—H2*A*⋯O1^i^	0.97	2.54	3.379 (2)	145
C9—H9⋯*Cg*^ii^	0.93	2.81	3.522 (2)	134
C12—H12⋯*Cg*^iii^	0.93	2.80	3.541 (2)	137

Symmetry codes: (i) 
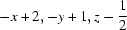
; (ii) 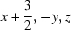; (iii) 
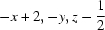
.

**Fig. 1.**
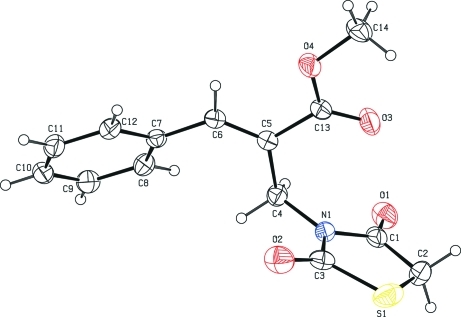
The molecular structure of the title compound with the atom numbering scheme. Displacement ellipsoids are drawn at the 30% probability level. H atoms are presented as a small spheres of arbitrary radius.

**Fig. 2.**
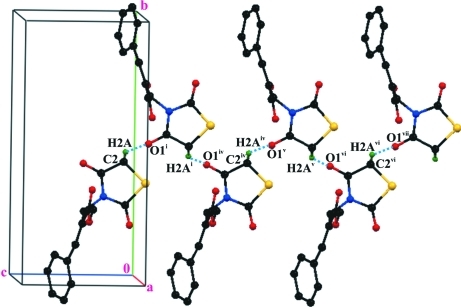
Part of the crystal structure of the title compound showing C—H···O intermolecular hydrogen bonds (dotted lines) generating C(4) chains along c axis. [Symmetry code: (i)2-x, 1-y, -1/2+z; (iv) x, y, -1+z; (v)2-x, 1-y, -3/2+z; (vi)x, y, -2+z; (vii)2-x, 1-y, -5/2+z].

**Fig. 3.**
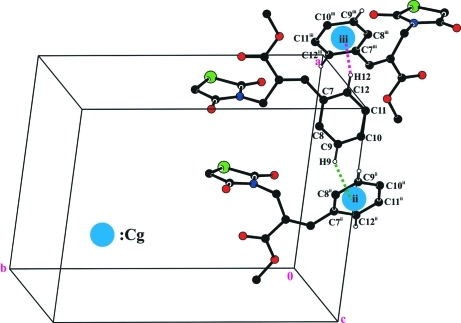
A view of the C—H···π interactions, in the molecular structure of the title compound. Cg is the centroid of the (C7–C12) benzene ring. [Symmetry code: (ii) 3/2-x, y, 1/2+z; (iii) 2-x, -y, -1/2+z.]

## supplementary crystallographic information

### Comment

Thiazolidine derivatives exhibit herbicidal (Chen *et al.,* 2000; Vicentini *et al.,* 1998), antineoplastic (Vigorita *et al.,* 1992), hypolipidemic (Jacop & Kutty, 2004) and anti-inflammatory (Kalia *et al.,* 2007) activities. In view of this importance, the crystal structure of the title compound has been carried out and the results are presented here.

Fig. 1. shows a displacement ellipsoid plot of (I), with the atom numbering scheme. The thiazolidine ring (S1/N1/C1–C3) is essentially planar [maximum deviation = 0.010 (2) Å for the C3 atom] and lies at an angle 60.1 (1)° with respect to the benzene ring. The significant difference in length of the C13—O4 = 1.333 (2) Å and C14—O4 = 1.445 (2) Å bonds is attributed to a partial contribution from the O^-^–C = O^+^–C resonance structure of the O3═C13—O4—C14 group (Merlino, 1971). This feature, commonly observed in the carboxylic ester group of the substituents in various compounds gives average values of 1.340 Å and 1.447 Å respectively for these bonds (Varghese *et al.,* 1986). The sum of bond angles around N1 (359°) indicates that N1 is in *sp*^2^ hybridization. The geometric parameters of the title molecule agrees well with those reported for similar structures (Fun *et al.,* 2009, Vijayakumar *et al.,* 2012).

In the crystal, intermolecular C—H···O hydrogen bonds invoving atoms C2 and O1 link molecules into C(4) chains running along *c* axis (Fig. 2). The crystal packing is further stabilized by C—H···π interactions, the first one between a benzene H atom and the benzene ring (C7–C12) of an adjacent molecule, with a C9—H9···Cg^ii^ seperation of 2.81 Å and the second one between a benzene H atom and the benzene ring (C7–C12) of a neighbouring molecule, with a C12—H12···Cg^iii^ seperation of 2.80 Å ( Table 1 and Fig. 3; Cg is the centroid of the C7–C12 benzene ring , symmetry code as in Fig. 3).

### Experimental

A solution of thiazolidine-2,4-dione (1 mmol, 0.117 g) and potassium carbonate (1.5 mmol, 0.207 g) in acetonitrile solvent was stirred for 15 minutes at room temperature. To this solution, methyl (2*Z*)-methyl-2 -(bromomethyl)-3-phenylprop-2-enoate (1 mmol, 0.254 g) was added dropwise till the addition is complete. After the completion of the reaction, as indicated by TLC, acetonitrile was evaporated. Ethyl acetate (15 ml) and water (15 ml) were added to the crude mass. The organic layer was dried over anhydrous sodium sulfate. Removal of solvent led to the crude product, which was purified through pad of silica gel (100–200 mesh) using ethylacetate and hexanes (1:9) as solvents. The pure title compound was obtained as a colourless solid (0.285 g, 98% yield). Recrystallization was carried out using ethylacetate as solvent.

### Refinement

H atoms were positioned geometrically, with C—H = 0.93–0.97 Å and constrained to ride on their parent atom, with *U*_iso_(H)=1.5*U*_eq_ for methyl H atoms and 1.2*U*_eq_(C) for other H atoms.

### Crystal data

**Table:**

C_14_H_13_NO_4_S	*F*(000) = 608
*M**_r_* = 291.31	*D*_x_ = 1.425 Mg m^−^^3^
Orthorhombic, *P**c**a*2_1_	Mo *K*α radiation, λ = 0.71073 Å
Hall symbol: P 2c -2ac	Cell parameters from 2955 reflections
*a* = 11.9274 (3) Å	θ = 1.3–26.9°
*b* = 15.6064 (6) Å	µ = 0.25 mm^−^^1^
*c* = 7.2949 (3) Å	*T* = 293 K
*V* = 1357.90 (8) Å^3^	Block, colourless
*Z* = 4	0.26 × 0.22 × 0.18 mm

### Data collection

**Table:**

Bruker APEXII CCD diffractometer	2948 independent reflections
Radiation source: fine-focus sealed tube	2576 reflections with *I* > 2σ(*I*)
graphite	*R*_int_ = 0.026
Detector resolution: 10.0 pixels mm^-1^	θ_max_ = 27.0°, θ_min_ = 2.2°
ω scans	*h* = −9→15
Absorption correction: multi-scan (*SADABS*; Sheldrick, 1996)	*k* = −19→19
*T*_min_ = 0.937, *T*_max_ = 0.956	*l* = −9→9
13845 measured reflections	

### Refinement

**Table:**

Refinement on *F*^2^	Secondary atom site location: difference Fourier map
Least-squares matrix: full	Hydrogen site location: inferred from neighbouring sites
*R*[*F*^2^ > 2σ(*F*^2^)] = 0.030	H-atom parameters constrained
*wR*(*F*^2^) = 0.082	*w* = 1/[σ^2^(*F*_o_^2^) + (0.0474*P*)^2^ + 0.0813*P*] where *P* = (*F*_o_^2^ + 2*F*_c_^2^)/3
*S* = 1.03	(Δ/σ)_max_ = 0.001
2948 reflections	Δρ_max_ = 0.12 e Å^−^^3^
182 parameters	Δρ_min_ = −0.22 e Å^−^^3^
1 restraint	Absolute structure: Flack (1983), 1348 Friedel pairs
Primary atom site location: structure-invariant direct methods	Flack parameter: 0.01 (7)

### Special details

**Table:**

Geometry. All esds (except the esd in the dihedral angle between two l.s. planes) are estimated using the full covariance matrix. The cell esds are taken into account individually in the estimation of esds in distances, angles and torsion angles; correlations between esds in cell parameters are only used when they are defined by crystal symmetry. An approximate (isotropic) treatment of cell esds is used for estimating esds involving l.s. planes.
Refinement. Refinement of F^2^ against ALL reflections. The weighted R-factor wR and goodness of fit S are based on F^2^, conventional R-factors R are based on F, with F set to zero for negative F^2^. The threshold expression of F^2^ > 2sigma(F^2^) is used only for calculating R-factors(gt) etc. and is not relevant to the choice of reflections for refinement. R-factors based on F^2^ are statistically about twice as large as those based on F, and R- factors based on ALL data will be even larger.

### Fractional atomic coordinates and isotropic or equivalent isotropic displacement parameters (Å^2^)

**Table:**

	*x*	*y*	*z*	*U*_iso_*/*U*_eq_	
C1	0.88283 (12)	0.42078 (11)	0.3591 (3)	0.0453 (4)	
C2	0.88406 (18)	0.46494 (13)	0.1782 (3)	0.0656 (6)	
H2A	0.9463	0.5049	0.1725	0.079*	
H2B	0.8150	0.4967	0.1609	0.079*	
C3	0.89926 (13)	0.30148 (13)	0.1635 (3)	0.0476 (4)	
C4	0.90014 (12)	0.27637 (10)	0.4973 (3)	0.0414 (3)	
H4A	0.8444	0.2315	0.4854	0.050*	
H4B	0.8827	0.3088	0.6071	0.050*	
C5	1.01448 (11)	0.23548 (9)	0.5200 (2)	0.0358 (3)	
C6	1.02982 (13)	0.15517 (9)	0.5761 (2)	0.0371 (3)	
H6	1.1043	0.1377	0.5805	0.044*	
C7	0.94821 (12)	0.08968 (10)	0.6322 (2)	0.0351 (3)	
C8	0.84781 (14)	0.10827 (10)	0.7234 (2)	0.0415 (4)	
H8	0.8296	0.1648	0.7508	0.050*	
C9	0.77608 (14)	0.04342 (11)	0.7725 (2)	0.0474 (4)	
H9	0.7092	0.0564	0.8319	0.057*	
C10	0.80204 (15)	−0.04058 (11)	0.7348 (3)	0.0501 (4)	
H10	0.7525	−0.0840	0.7672	0.060*	
C11	0.90151 (15)	−0.06013 (11)	0.6491 (3)	0.0492 (4)	
H11	0.9195	−0.1170	0.6246	0.059*	
C12	0.97448 (14)	0.00418 (10)	0.5993 (2)	0.0421 (4)	
H12	1.0421	−0.0097	0.5431	0.050*	
C13	1.11324 (12)	0.29123 (10)	0.4825 (2)	0.0396 (3)	
C14	1.31055 (14)	0.30026 (12)	0.4659 (3)	0.0592 (5)	
H14A	1.3018	0.3385	0.3638	0.089*	
H14B	1.3737	0.2634	0.4448	0.089*	
H14C	1.3228	0.3330	0.5756	0.089*	
N1	0.89204 (9)	0.33305 (8)	0.3393 (2)	0.0387 (3)	
O1	0.87429 (10)	0.45658 (8)	0.5042 (2)	0.0606 (3)	
O2	0.90570 (11)	0.22625 (10)	0.1267 (2)	0.0704 (4)	
O3	1.10784 (9)	0.36635 (7)	0.4537 (2)	0.0627 (4)	
O4	1.21042 (8)	0.24908 (7)	0.4864 (2)	0.0562 (3)	
S1	0.89843 (4)	0.38495 (4)	0.00251 (8)	0.07068 (18)	

### Atomic displacement parameters (Å^2^)

**Table:**

	*U*^11^	*U*^22^	*U*^33^	*U*^12^	*U*^13^	*U*^23^
C1	0.0282 (7)	0.0397 (8)	0.0680 (12)	−0.0004 (6)	−0.0036 (8)	0.0022 (9)
C2	0.0571 (11)	0.0562 (12)	0.0834 (16)	−0.0031 (9)	−0.0105 (10)	0.0271 (11)
C3	0.0327 (8)	0.0616 (11)	0.0484 (10)	0.0003 (7)	−0.0045 (7)	−0.0026 (9)
C4	0.0379 (8)	0.0397 (8)	0.0467 (9)	0.0014 (6)	0.0036 (7)	0.0044 (8)
C5	0.0328 (7)	0.0388 (7)	0.0359 (7)	−0.0008 (5)	−0.0004 (6)	−0.0030 (6)
C6	0.0333 (7)	0.0420 (8)	0.0360 (7)	0.0000 (6)	−0.0017 (6)	−0.0004 (6)
C7	0.0354 (7)	0.0388 (8)	0.0312 (7)	0.0002 (6)	−0.0038 (6)	0.0016 (6)
C8	0.0456 (9)	0.0414 (8)	0.0376 (8)	0.0038 (6)	0.0035 (7)	0.0025 (7)
C9	0.0416 (9)	0.0605 (10)	0.0401 (9)	−0.0026 (7)	0.0059 (8)	0.0073 (7)
C10	0.0526 (9)	0.0543 (10)	0.0434 (9)	−0.0163 (8)	−0.0092 (8)	0.0097 (8)
C11	0.0621 (11)	0.0373 (8)	0.0482 (10)	−0.0015 (7)	−0.0080 (9)	0.0020 (7)
C12	0.0442 (9)	0.0401 (8)	0.0419 (9)	0.0055 (7)	−0.0023 (7)	0.0022 (7)
C13	0.0374 (7)	0.0409 (8)	0.0405 (8)	−0.0027 (6)	−0.0025 (7)	−0.0052 (7)
C14	0.0339 (8)	0.0728 (11)	0.0709 (14)	−0.0108 (8)	0.0018 (9)	0.0034 (11)
N1	0.0350 (6)	0.0353 (7)	0.0457 (8)	−0.0001 (5)	−0.0021 (6)	0.0020 (6)
O1	0.0543 (7)	0.0463 (7)	0.0811 (10)	0.0042 (5)	−0.0015 (8)	−0.0153 (8)
O2	0.0777 (10)	0.0645 (9)	0.0690 (10)	0.0068 (7)	−0.0084 (8)	−0.0233 (8)
O3	0.0443 (7)	0.0381 (6)	0.1056 (13)	−0.0058 (5)	0.0005 (7)	0.0012 (7)
O4	0.0319 (5)	0.0495 (6)	0.0873 (9)	−0.0023 (4)	0.0024 (6)	0.0087 (7)
S1	0.0586 (3)	0.1007 (4)	0.0527 (3)	0.0051 (2)	−0.0039 (3)	0.0233 (3)

### Geometric parameters (Å, °)

**Table:**

C1—O1	1.202 (3)	C7—C12	1.392 (2)
C1—N1	1.381 (2)	C7—C8	1.400 (2)
C1—C2	1.489 (3)	C8—C9	1.373 (2)
C2—S1	1.797 (3)	C8—H8	0.9300
C2—H2A	0.9700	C9—C10	1.375 (2)
C2—H2B	0.9700	C9—H9	0.9300
C3—O2	1.207 (2)	C10—C11	1.375 (3)
C3—N1	1.376 (2)	C10—H10	0.9300
C3—S1	1.754 (2)	C11—C12	1.377 (3)
C4—N1	1.456 (2)	C11—H11	0.9300
C4—C5	1.515 (2)	C12—H12	0.9300
C4—H4A	0.9700	C13—O3	1.1928 (19)
C4—H4B	0.9700	C13—O4	1.3330 (18)
C5—C6	1.331 (2)	C14—O4	1.4445 (18)
C5—C13	1.490 (2)	C14—H14A	0.9600
C6—C7	1.470 (2)	C14—H14B	0.9600
C6—H6	0.9300	C14—H14C	0.9600
			
O1—C1—N1	124.04 (18)	C9—C8—H8	119.9
O1—C1—C2	124.52 (16)	C7—C8—H8	119.9
N1—C1—C2	111.44 (18)	C8—C9—C10	120.69 (16)
C1—C2—S1	108.14 (13)	C8—C9—H9	119.7
C1—C2—H2A	110.1	C10—C9—H9	119.7
S1—C2—H2A	110.1	C9—C10—C11	119.78 (15)
C1—C2—H2B	110.1	C9—C10—H10	120.1
S1—C2—H2B	110.1	C11—C10—H10	120.1
H2A—C2—H2B	108.4	C10—C11—C12	120.23 (16)
O2—C3—N1	124.05 (18)	C10—C11—H11	119.9
O2—C3—S1	124.99 (16)	C12—C11—H11	119.9
N1—C3—S1	110.95 (14)	C11—C12—C7	120.73 (16)
N1—C4—C5	113.71 (13)	C11—C12—H12	119.6
N1—C4—H4A	108.8	C7—C12—H12	119.6
C5—C4—H4A	108.8	O3—C13—O4	122.40 (13)
N1—C4—H4B	108.8	O3—C13—C5	124.31 (13)
C5—C4—H4B	108.8	O4—C13—C5	113.29 (13)
H4A—C4—H4B	107.7	O4—C14—H14A	109.5
C6—C5—C13	119.85 (13)	O4—C14—H14B	109.5
C6—C5—C4	123.64 (13)	H14A—C14—H14B	109.5
C13—C5—C4	116.47 (12)	O4—C14—H14C	109.5
C5—C6—C7	130.50 (15)	H14A—C14—H14C	109.5
C5—C6—H6	114.8	H14B—C14—H14C	109.5
C7—C6—H6	114.8	C3—N1—C1	117.22 (16)
C12—C7—C8	118.24 (14)	C3—N1—C4	121.05 (13)
C12—C7—C6	118.01 (14)	C1—N1—C4	121.65 (16)
C8—C7—C6	123.69 (14)	C13—O4—C14	116.35 (12)
C9—C8—C7	120.28 (15)	C3—S1—C2	92.23 (10)
			
O1—C1—C2—S1	179.94 (13)	C6—C5—C13—O4	8.7 (2)
N1—C1—C2—S1	−0.45 (17)	C4—C5—C13—O4	−173.45 (16)
N1—C4—C5—C6	−142.14 (16)	O2—C3—N1—C1	−178.70 (15)
N1—C4—C5—C13	40.1 (2)	S1—C3—N1—C1	1.56 (16)
C13—C5—C6—C7	175.87 (15)	O2—C3—N1—C4	4.6 (2)
C4—C5—C6—C7	−1.8 (3)	S1—C3—N1—C4	−175.10 (10)
C5—C6—C7—C12	149.04 (17)	O1—C1—N1—C3	178.90 (14)
C5—C6—C7—C8	−33.6 (3)	C2—C1—N1—C3	−0.71 (18)
C12—C7—C8—C9	−2.2 (2)	O1—C1—N1—C4	−4.5 (2)
C6—C7—C8—C9	−179.56 (15)	C2—C1—N1—C4	175.93 (14)
C7—C8—C9—C10	0.7 (3)	C5—C4—N1—C3	66.50 (18)
C8—C9—C10—C11	0.8 (3)	C5—C4—N1—C1	−110.01 (16)
C9—C10—C11—C12	−0.7 (3)	O3—C13—O4—C14	4.0 (3)
C10—C11—C12—C7	−1.0 (3)	C5—C13—O4—C14	−175.17 (15)
C8—C7—C12—C11	2.4 (2)	O2—C3—S1—C2	178.77 (16)
C6—C7—C12—C11	179.87 (15)	N1—C3—S1—C2	−1.50 (13)
C6—C5—C13—O3	−170.43 (18)	C1—C2—S1—C3	1.09 (14)
C4—C5—C13—O3	7.4 (3)		

### Hydrogen-bond geometry (Å, °)

**Table:**

Cg is the centroid of the C7–C12 benzene ring.

**Table:**

*D*—H···*A*	*D*—H	H···*A*	*D*···*A*	*D*—H···*A*
C2—H2A···O1^i^	0.97	2.54	3.379 (2)	145.
C9—H9···Cg^ii^	0.93	2.81	3.522 (2)	134.
C12—H12···Cg^iii^	0.93	2.80	3.541 (2)	137.

Symmetry codes: (i) −*x*+2, −*y*+1, *z*−1/2; (ii) *x*+3/2, −*y*, *z*; (iii) −*x*+2, −*y*, *z*−1/2.

## References

[bb1] Bruker (2004). *APEX2*, *SAINT* and *XPREP* Bruker AXS Inc., Madison, Wisconsin, USA.

[bb2] Chen, H. S., Li, Z. M. & Han, Y. F. (2000). *J. Agric. Food Chem.* **48**, 5312–5315. 10.1021/jf991065s 11087478

[bb3] Farrugia, L. J. (1997). *J. Appl. Cryst.* **30**, 565.

[bb4] Flack, H. D. (1983). *Acta Cryst.* A**39**, 876–881.

[bb5] Fun, H.-K., Goh, J. H., Vinayaka, A. C. & Kalluraya, B. (2009). *Acta Cryst.* E**65**, o2094. 10.1107/S160053680903027X PMC2970021 21577510

[bb6] Jacop, J. & Kutty, G. N. (2004). *Indian Drugs*, **41**, 76–79.

[bb7] Kalia, R., Rao, C. M. & Kutty, N. G. (2007). *Arzneim. Forsch. (Drug Res.)*, **57**, 616–622. 10.1055/s-0031-1296657 17966761

[bb8] Merlino, S. (1971). *Acta Cryst.* B**27**, 2491–2492.

[bb9] Sheldrick, G. M. (1996). *SADABS* University of Göttingen, Germany.

[bb10] Sheldrick, G. M. (2008). *Acta Cryst.* A**64**, 112–122. 10.1107/S0108767307043930 18156677

[bb11] Spek, A. L. (2009). *Acta Cryst.* D**65**, 148–155. 10.1107/S090744490804362X PMC2631630 19171970

[bb12] Varghese, B., Srinivasan, S., Padmanabhan, P. V. & Ramadas, S. R. (1986). *Acta Cryst.* C**42**, 1544–1546.

[bb13] Vicentini, C. B., Manfrini, M., Veronese, A. C. & Guarneri, M. (1998). *J. Heterocycl. Chem.* **35**, 29–36.

[bb14] Vigorita, M. G., Basile, M., Zappala, C., Gabbrielli, G. & Pizzimenti, F. (1992). *Farmaco*, **47**, 893–906. 1388607

[bb15] Vijayakumar, S., Murugavel, S., Kannan, D. & Bakthadoss, M. (2012). *Acta Cryst.* E**68**, o156–o157. 10.1107/S1600536811053682 PMC3254499 22259442

